# Cross Talk between Neuroregulatory Molecule and Monocyte: Nerve Growth Factor Activates the Inflammasome

**DOI:** 10.1371/journal.pone.0121626

**Published:** 2015-04-15

**Authors:** Ananya Datta-Mitra, Smriti Kundu-Raychaudhuri, Anupam Mitra, Siba P. Raychaudhuri

**Affiliations:** 1 Division of Rheumatology, Allergy and Clinical Immunology, University of California Davis, School of Medicine, Davis, CA, 95616, United States of America; 2 VA Medical Center Sacramento, Mather, CA, 95655, United States of America; 3 Department of Dermatology, University of California Davis, School of Medicine, Sacramento, CA, 95817, United States of America; University Paris Sud, FRANCE

## Abstract

**Background:**

Increasing evidence points to a role for the extra-neuronal nerve growth factor (NGF) in acquired immune responses. However, very little information is available about its role and underlying mechanism in innate immunity. The role of innate immunity in autoimmune diseases is becoming increasingly important. In this study, we explored the contribution of pleiotropic NGF in the innate immune response along with its underlying molecular mechanism with respect to IL-1β secretion.

**Methods:**

Human monocytes, null and NLRP3 deficient THP-1 cell lines were used for this purpose. We determined the effect of NGF on secretion of IL-1β at the protein and mRNA levels. To determine the underlying molecular mechanism, the effect of NGF on NLRP1/NLRP3 inflammasomes and its downstream key protein, activated caspase-1, were evaluated by ELISA, immunoflorescence, flow cytometry, and real-time PCR.

**Results:**

In human monocytes and null THP-1 cell line, NGF significantly upregulates IL-1β at protein and mRNA levels in a caspase-1 dependent manner through its receptor, TrkA. Furthermore, we observed that NGF induces caspase-1 activation through NLRP1/NLRP3 inflammasomes, and it is dependent on the master transcription factor, NF-κB.

**Conclusions:**

To best of our knowledge, this is the first report shedding light on the mechanistic aspect of a neuroregulatory molecule, NGF, in innate immune response, and thus enriches our understanding regarding its pathogenic role in inflammation. These observations add further evidence in favor of anti-NGF therapy in autoimmune diseases and also unlock a new area of research about the role of NGF in IL-1β mediated diseases.

## Introduction

Innate immune response is initiated by the interaction of pattern recognition receptors (PRRs) in immune cells with either microbial pathogen associated molecular patterns (PAMPs) or cellular damage associated molecular patterns (DAMPs), resulting in the release of pro-inflammatory cytokines [1, 2]. Among multiple germ-line encoded PRRs, the nod-like receptor (NLR) proteins trigger the innate immune response through formation of the 'inflammasome' complex in order to tackle the PAMPs and DAMPs [1, 2]. The 'inflammasome' is a large, multiprotein complex, comprised of NLR protein, an adapter protein, and pro-caspase-1 [2–5]. NLRP1 and NLRP3 inflammasomes are so far the best characterized [4, 6]. Although there are some structural differences between NLRP1 and NLRP3 inflammasomes, the activation process is similar [4]. Briefly, in the presence of exogenous or endogenous stimuli, conformational changes in the NLRPs lead to the recruitment of procaspase-1, resulting in active caspase-1 formation. This activation of caspase-1, through autoproteolytic maturation, leads to the processing and secretion of the proinflammatory cytokines interleukin-1β (IL-1β) and IL-18 [1, 4, 7–9]. IL-1β is a pleiotropic cytokine secreted chiefly by myeloid cells that further induces the secretion of other proinflammatory cytokines and antimicrobial proteins, thereby boosting host innate immune responses [7, 10, 11]. In addition to the innate immune response, the role of IL-1β has been well established in the differentiation of pathogenic Th17 cells and in different autoimmune diseases including rheumatoid arthritis (RA) and psoriatic diseases [12–18].

Several *in vivo* and *in vitro* studies establish the extra-neuronal role of nerve growth factor (NGF) in autoimmune diseases [19–21] and illustrate the contribution of NGF in the acquired immune response. It has been established that the immune cells such as T and B lymphocytes, dendritic cells and monocytes/macrophages express NGF and its receptors tyrosine kinase A (TrkA) and p75-neurotrophin receptor (p75-NTR) [22]. TrkA is specific for NGF and its expression is essential for NGF function. p75-NTR binds to all neurotrophins and increases TrKA affinity for neurotrophins [23]. Although some information is available on TrkA signaling in immune cells, still there is need for further investigations [19, 24].

In the last few years, increasing evidence strengthens the importance of the innate immune response in the pathogenesis of autoimmune diseases [25–31]. In this context, the contribution of the ‘inflammasome’, a fundamental component of innate immunity, has been shown in autoimmune diseases [29, 32–36]. So far, the role of NGF in the innate immune response remains unexplored except one study in mid 90s, which reported that NGF induces IL-1β secretion in murine macrophages but did not provide the underlying mechanistic insight [37]. Here, we have explored the regulatory role of NGF in the human innate immune response by measuring IL-1β, and further dissected out the underlying molecular mechanism. We observed that NGF activates NLRP1 and NLRP3 inflammasomes and the key cysteine protease, caspase-1, through its receptor TrkA, resulting in the release of mature IL-1β from both monocytes and THP-1 cells, a monocyte cell line.

## Materials and Methods

### Ethics statement

This study was approved by the Institutional Review Board (IRB) of the VA Sacramento Medical Center. IRB approved consent forms were signed by the participants and venous blood was collected by phlebotomists.

### Chemicals

Human NGF-β (Calbiochem, San Diego, CA, USA), K252a (Axxora LLC, Farmingdale, NY, USA), and PLX7486 (TrkA inhibitor, kind gift from Plexxikon, Berkeley, CA, USA) were used at 100 ng/ml, 100 ng/ml and 1 μM respectively. LPS (Sigma, St. Louis, MO, USA), Ac-YVAD-CHO, Caspase-1 inhibitor [38] (YVAD, EMD Millipore, Billerica, MA,USA), and pyrrolidine-dithio-carbamate (PDTC), NF-κB inhibitor (Sigma) [39] were used at 1 μg/ml, 50μM, and 50μM respectively. Anti-p75-NTR rabbit anti-mouse polyclonal antibody (EMD Millipore, AB1554) was used in 1:1000 dilution (1μg/ml as per manufacturer’s instruction) for blocking p75-NTR as per manufacturer’s instructions. The p75-NTR blocking antibody is a transmembrane glycoprotein consisting of an extracellular domain responsible for ligand binding.

### Cell culture

***\***Monocytes from human subjects (n = 12) were magnetically sorted from peripheral blood using a CD14 positive selection kit (Stem Cell Tech, Cat. #18088, Vancouver, BC, Canada) as per manufacturer’s instructions. The purity (>85%) was confirmed by flow cytometry. NLRP3 deficient THP-1 cells were purchased from Invivogen (THP1-defNLRP3, Invivogen, San Diego, CA, USA) and cultured as per manufacturer’s instructions. 1x10^6^ monocytes or THP-1 cells (ATCC TIB 202) or THP1-defNLRP3 (DNLRP3) were treated with LPS, NGF, K252a, PLX7486, YVAD, anti-p75-NTR and PDTC alone or in combination in culture plates. All inhibitors were added 1 hr prior to NGF treatment and the cells were incubated for 24 hours. Culture medium were collected from differently treated samples by centrifugation and subjected to IL-1β and Caspase-1 ELISA (R&D Systems). In addition, activated caspase-1 was also evaluated by flow cytometry (FACSCalibur, BD Biosciences, San Jose, CA, USA) and fluorescence microscopy (IF) using a caspase-1 carboxyfluorescein-YVAD-fluoromethylketone, a fluorescent labeled inhibitor of caspase (FLICA) kit (Immunochemistry Technologies, Bloomington, MN, USA) following the manufacturer’s protocol [40, 41]. Briefly, the FLICA reagent freely permeates into the cell, covalently binds to active caspase-1, and remains inside the cell while the unbound FLICA diffuses out of the cell and is washed away. The fluorescent signal (FLICA^+^) is a direct measure of intracellular active caspase-1 enzyme activity and the fluorescent signal can be detected by immunocytochemistry and flow cytometry [42]. In IF, cells showing active caspase-1 were manually counted in five different fields of view for each treatment by two independent observers. Analysis of flow cytometry data showing number of FLICA positive cells was done using FlowJo software (Tree Star Inc, Ashland, OR, USA).

### Western blot and real-time PCR

Another experiment was set up in similar conditions, protein and total RNA was extracted for western blot and quantitative real-time PCR respectively, using TRIzol reagent (Invitrogen, Grand Island, NY, USA) [43]. 25μg protein for each lysate was subjected to SDS-PAGE as per our standardized protocol [43, 44] with specific primary antibodies (Cell signaling, Danvers, MA, USA) for phospho NF-κB p65 (Cat.#3033), total NF-κB (Cat.#8242), TrkA (Santa Cruz Biotechnology, Dallas, TX, USA, Cat.# sc-118), p75-NTR (Cat.# 2693), α-tubulin (Cat.#2125), and bands were analyzed using Image J software (NIH). Real-time PCR was conducted with extracted total cellular RNA using SYBR green (Qiagen, Valencia, CA, USA) and specific primers for IL1B (Forward: 5’TTCGACACATGGGATAACGA3’ Reverse: 5’TCTTTCAACACGCAGGACAG3’), CASP1 (Forward:5’TACAGAGCTGGAGGCATTTG3’ Reverse: 5’GATCACCTTCGGTTTGTCCT3’), NLRP1 (Forward: 5’TGCCTCACTCCTCTACCAAG3’ Reverse: 5’AATTCCTGACGTTTCATCCA3’), NLRP3 (Forward: 5’GAAGAGGAGTGGATGGGTTT3’ Reverse: 5’CGTGTGTAGCGTTTGTTGAG3’), and RN18S1 (Forward: 5’ TCAAGAACGAAAGTCGGAGG3’ Reverse: 5’GGACATCTAAGGGCATCACA3’). Analysis of relative gene expression was performed as described in earlier publications [43, 45].

### Statistical analysis

All experiments were done in triplicate and results expressed as Mean±SEM (Standard Error of Mean). Statistical analysis was done using GraphPad Prism software, version 5.0 (La Jolla, CA, USA). Non parametric unpaired tests (Mann-Whitney U Test, Kruskal-Wallis one-way analysis of variance by ranks) were used to determine statistical significance. A p value of <0.05 was considered statistically significant.

## Results and Discussion

### NGF/TrkA interaction induces mature IL-1β secretion in human monocytic cell line

**Fig 1:**
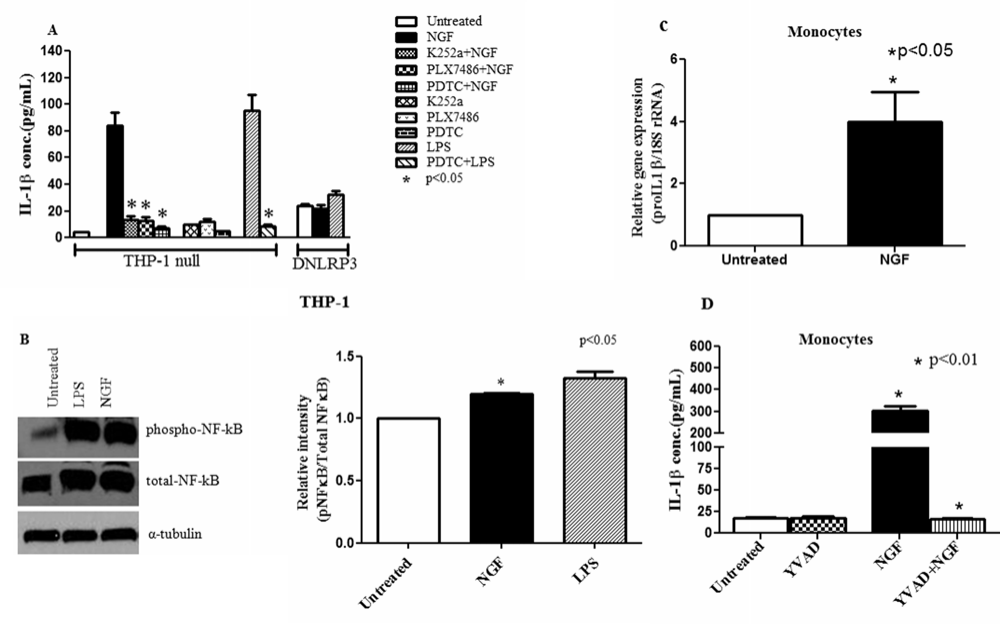
NGF/TrkA interaction induces IL-1β at protein and mRNA level in monocytic cell line through NF-κB. **(A)** In null THP-1 cells, NGF (100 ng/ml) significantly induced mature IL-1β secretion compared to NLRP3 deficient THP-1 cells (DNLRP3). Prior treatment with TrkA inhibitors (K252a, 100 ng/ml and PLX7486, 100 ng/ml) or NF-κB inhibitor, PDTC (50 μM) effectively inhibited NGF induced IL-1β release (n = 6). **(B)** Western blot assay was done with null THP-1 cells cultured for 24 hrs with NGF (100 ng/ml) and LPS (1 μg/ml). Representative immunoblot and bar diagram (n = 6) showing significant phosphorylation of NF-κB p65 with NGF. LPS was used as a positive control. **(C)** Real-time PCR was performed with total RNA extracted from human monocytes cultured for 24 hrs with NGF (n = 8). NGF significantly upregulated IL-1β mRNA. **(D)** Magnetically sorted monocytes (n = 12) were cultured with or without NGF (100 ng/ml) and caspase-1 inhibitor (YVAD, 50 μM). Bar diagram showing significant induction of IL-1β with NGF and YVAD effectively blocked it. Data expressed as Mean±SEM. Mann Whitney U test was done to determine statistical significance.

**Fig 2:**
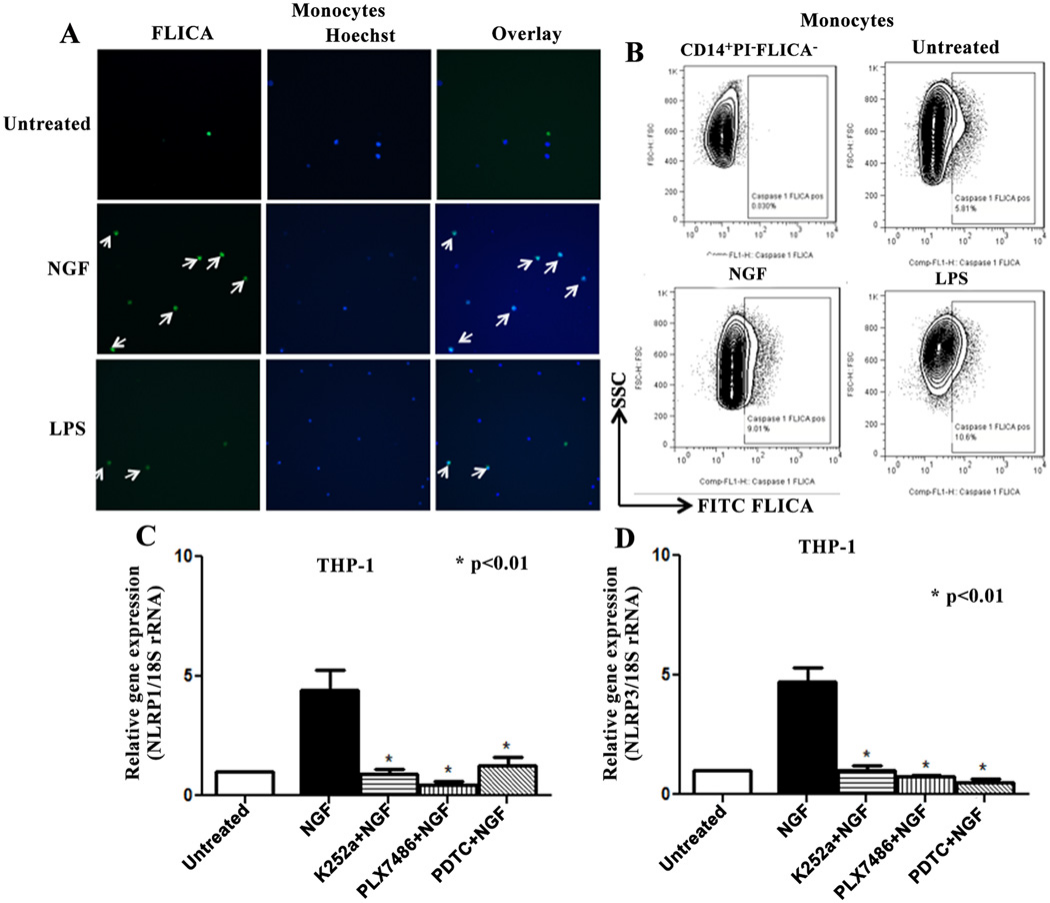
NGF induces activation of caspase-1 through NLRP1/NLRP3 inflammasomes in monocytic cell line. **(A)** Magnetically sorted human monocytes (n = 8) were cultured 24 hrs with NGF (100 ng/ml) and activated caspase-1 was determined using FLICA kit. Representative immunofluorescent images (20x, Leica Microscope) showing more FLICA^+^ positive monocytes in NGF treated culture compared to untreated. **(B)** Monocytes cultured in similar settings with FLICA were subjected to flow cytometry to determine activated caspase-1 using FlowJo. First representative histogram is the stain-1 for FLICA using PI^-^ cells. NGF treatment showed more FLICA^+^ monocytes compared to untreated control. **(C)** Real-time PCR was done with extracted total RNA from null THP-1 cells cultured 24 hrs with or without NGF (100 ng/ml), K252a (100 ng/ml), PLX7486 (100 ng/ml) and PDTC (50 μM). 18S rRNA was used as an endogenous control. NGF significantly upregulated mRNA of NLRP1, which is effectively blocked by K252a, PLX7486 and PDTC (n = 6). **(D)** In similar setting, NGF/TrkA interaction significantly upregulated mRNA of NLRP3 in a NF-κB dependent manner (n = 6). Data expressed as Mean ± SEM. Mann Whitney U test was done to determine statistical significance.

Monocytes/macrophages are the major source of IL-1β and play a crucial role in maintaining the host innate immune response. To address the regulatory role of NGF in the innate immune response, we first evaluated the effect of NGF on IL-1β secretion in THP-1, a human monocytic cell line. Using ELISA, we observed significant induction of IL-1β expression and secretion with NGF treatment (206.4±16.81pg/ml, p<0.001) compared to the untreated (19.08±1.78 pg/ml) group, which is consistent with the only available previous report [37]. To further confirm this observation, we treated NLRP3 deficient THP-1 (DNLRP3) and null THP-1 cells with NGF and determined the IL-1β secretion. NGF treatment could not induce IL-1β secretion in DNLRP3 cells (21.58±2.7 pg/ml) compared to untreated DNLRP3 cells (23.6±1.6 pg/ml) (Fig 1A). The TrkA inhibitors (K252a: 15.07±1.81 pg/ml, p<0.001 and PLX7486: 14.52±2.09 pg/ml, p<0.001) effectively blocked the NGF mediated increase of IL-1β (Fig 1A). Apart from inhibiting phosphorylation of TrkA, K252a, a fungal alkaloid, also inhibits protein kinase C [46]. Thus, to confirm the inhibitory effect of specific NGF/TrkA interaction, a novel TrkA inhibitor, PLX7486, was used. LPS served as a positive control. In line with previous studies, we also found that monocytes and THP-1 cells express both TrkA and p75-NTR (data not shown), however, blocking p75-NTR had insignificant effect on NGF-induced IL-1β secretion (NGF: 206.4±16.81 pg/ml vs. NGF+p75-NTR antibody: 218.2±6.99 pg/ml) in the THP-1 cells, which confirms that NGF induced IL-1β secretion is dependent on NGF/TrkA interaction. Whereas, in a recent publication Prencipe G *et al*. investigated whether NGF can induce IL-1β secretion in LPS activated monocytes and they did not find any increase in IL-1β levels in the monocytes treated with NGF and LPS [47]. Our results are more relevant. Here, we have explored the regulatory role of NGF in the human innate immune response by directly treating the monocytes and the THP-1 cell lines with NGF. LPS treated human monocytes were used as positive controls. Further, we have demonstrated that NGF induced IL-1β secretion is mediated through Caspase 1 activation which cleaves pro-IL-1β to form activated IL-1β (Fig 2).

### NGF induced IL-1β secretion is regulated at the transcriptional level through NF-κB

The release of mature IL-1β is tightly regulated both at transcriptional and posttranscriptional levels [40, 48]. To elucidate the underlying molecular mechanism of NGF induced mature IL-1β secretion, we first evaluated the effect of NGF at the transcriptional level. For this, we chose NF-κB, as previous studies have shown that NF-κB is an important transcription factor for several cytokines including IL-1β [49, 50]. We used the NF-κB inhibitor PDTC (pyrrolidine-dithio-carbamate) [39, 51] and observed that this effectively blocked the NGF induced mature IL-1β secretion in the THP-1 cell line (Fig 1A). This suggests that the NGF-induced mature IL-1β secretion is regulated at a transcriptional level in part through NF-κB. To confirm this observation, we performed western blot with the lysates of NGF treated THP-1 cells. NGF significantly upregulates total NF-κB as compared to untreated control (1.2 ± 0.01 vs 1, p<0.05). It also induces marked effect on the phosphorylation of NF-κB p65 (Fig 1B). This results in increased transcriptional activity of NF-κB, which initiates the transcription of several cytokines including IL-1β [49, 50]. These observations support the notion that NGF regulates mature IL-1β release at a transcriptional level in part through NF-κB.

### NGF upregulates the transcription of IL-1β

We wanted to see whether NGF can upregulate IL-1β transcription (proIL-1β) also. To substantiate this, we performed real-time PCR in magnetically sorted human monocytes. We observed that NGF induced a marked upregulation of proIL-1β (4±0.9 fold, p<0.05) compared to untreated cells (Fig 1C).

### NGF induced IL-1β secretion is regulated at posttranscriptional level through caspase-1

As release of mature IL-1β is critically regulated at post-transcriptional level through the key cysteine protease, caspase-1 (IL-1β converting enzyme), our next objective was to determine whether NGF contributes to the posttranscriptional modification of IL-1β. To achieve this, we determined the effect of NGF on activation of caspase-1 in the monocytic cell line. As determined by ELISA of the culture supernatants, NGF significantly induced active caspase-1 (78.7±12.12 pg/ml, p<0.05) in human monocytes compared to untreated controls (40±4.8 pg/ml). This observation was confirmed in NLRP3 deficient THP-1 cells. In NLRP3 deficient THP-1 cells, NGF could not induce significant caspase-1 production compared to THP-1 null cells (THP-1-defNLRP3: 62.7±0.7 pg/ml vs. THP-1 null: 78.7±12.12 pg/ml). We also sought the expression of NGF induced caspase-1 in human monocytes by ELISA, immunocytochemistry and flow cytomtery [40, 41]. In ELISA, the caspase-1 inhibitor YVAD [38] effectively blocked the NGF induced IL-1β secretion (Fig 1D). In the immunocytochemical assay, NGF treated human monocytes showed more FLICA^+^ cells (18%) compared to untreated controls (4%) (Fig 2A). A similar trend was observed in the THP-1 cell line (NGF: 9% FLICA^+^ cells; untreated: 3% FLICA^+^ cells). These observations are in line with the caspase-1 ELISA findings and indicate the upregulation of activated caspase-1 in monocytic cells line by NGF. LPS was used as a positive control. To confirm the immunocytochemical observations, similar experiments were repeated and assessed by flow cytometry using FlowJo software. Live (PI^-^) CD14^+^ monocytes were gated for analysis. NGF treated monocytes were more FLICA^+^ compared to untreated controls (Fig 2B). These observations strongly suggest that NGF induces the activation of caspase-1 in the human monocytic cell line.

Next, we wanted to elucidate whether NGF also induced the upregulation of procaspase-1 transcription, similar to what NGF did for IL-1β. Using real-time PCR (THP-1 cells, n = 6, each experiment done in triplicates), we observed a marked upregulation of procaspase-1 mRNA with NGF (CASP1, 6.1±1.1 fold, p<0.01) compared to the untreated control. In addition, TrkA inhibitors (K252a, 0.28±0.13 fold, p<0.01 and PLX7486, 2.4±1.3 fold, p<0.05) as well as the NF-κB inhibitor (PDTC, 2.7±0.84 fold, p<0.05) effectively blocked this effect. Thus, the NGF/TrkA interaction induced the activation of caspase-1 by upregulating procaspase-1 transcription. Taken together, these observations confirm that the NGF/TrkA interaction induces mature IL-1β in a human monocytic cell line by (i) upregulating transcription of proIL-1β, procaspase-1 through NF-κB; and (ii) modulating posttranscriptional proteolytic cleavage through active caspase-1. Our observations are consistent with previous studies, which showed that IL-1β is tightly controlled at transcriptional and posttranscriptional levels: exogenous/endogenous stimuli regulate proIL-1β transcription, whereas secretion of mature IL-1β is modulated by 'inflammasomes' [4, 40].

### NGF induces caspase-1 activation through NLRP1 and NLRP3 inflammasomes

Our next aim was to explore the molecular mechanism behind the activation of caspase-1 by NGF. In the presence of exogenous/endogenous stimuli, a conformational change in the sensor protein (NLRP) leads to recruitment of procaspase-1 through the CARD domain and subsequent formation of active caspase-1. During formation of the crucial ‘inflammasome’ complex, we have shown that the NGF/TrkA interaction upregulates procaspase-1 through NF-κB. Our next objective was to elucidate the contribution of NGF in upstream components of the ‘inflammasome’ complex, such as NLRP1 and NLRP3. Activation of the NLRP3 inflammasome is determined by its transcriptional induction [40]. We evaluated the effect of NGF on NLRP1 and NLRP3 mRNA in THP-1 cells in an attempt to dissect out the underlying molecular mechanism. In real-time PCR, we found significant upregulation of NLRP1 (4.43±0.82 fold, p<0.01, Fig 2C) and NLRP3 mRNA (4.7±0.63 fold, p<0.01, Fig 2D) with NGF. TrkA inhibitors (K252a and PLX7486) as well as a NF-κB inhibitor (PDTC) effectively blocked NGF mediated upregulation of NLRP1 and NLRP3 genes. A previous study also showed that NLRP3 inflammasome activation is dependent on NF-κB [40]. These observations suggest that the NGF/TrkA interaction induces NLRP1 and NLRP3 inflammasomes through NF-κB. In this context, previous studies have shown that the NGF/TrkA interaction leads to activation of the PI3K/Akt pathway [52, 53]. Moreover, activation of Akt leads to the activation of NF-κB [54]. However, we have not done any experiments in this study to show whether the NGF mediated upregulation of NF-κB is dependent on PI3K/Akt pathway or not.

**Fig 3:**
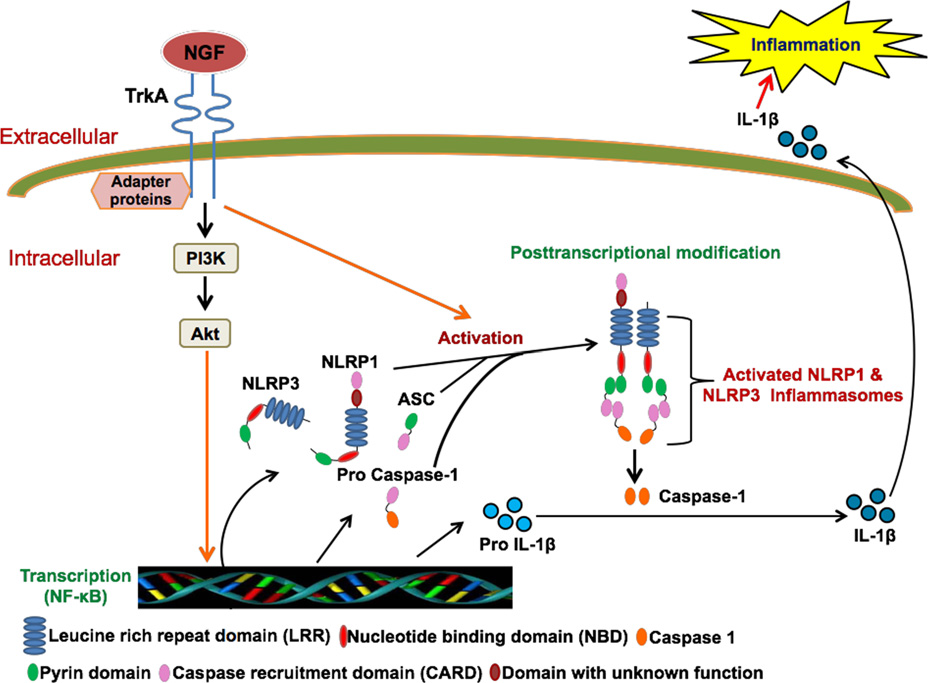
Schematic diagram representing the NGF-induced activation of inflammasome complex and subsequent release of mature IL-1β. Briefly, NGF/TrkA interaction modulates the IL-1β release by acting at both transcriptional and posttranscriptional level. At transcriptional level, NGF upregulates the mRNA expression of NLRP1, NLRP3, procaspase-1 and proIL-1β. By upregulating the NLRP1/NLRP3 mRNA, NGF induces formation of active inflammasome complex, which converts pro IL-1β to mature IL-1β.

Taken together, we conclude that the pleiotropic NGF/TrkA interaction contributes to the host innate immune response by modulating mature IL-1β secretion in monocytic cell lines both at transcriptional and posttranscriptional levels through the key cysteine protease, caspase-1, and its upstream inflammasomes (Fig 3). In addition to our existing knowledge regarding the extra-neuronal role of NGF in acquired immune responses, we have uncovered a novel cellular-molecular mechanism through which NGF regulates the human innate immune response. This study enhances the understanding of the contribution of the pleiotropic NGF in human inflammatory diseases at a molecular level and provides strong support to develop NGF targeted therapies for autoimmune diseases [19, 55].
